# The School Doctor and School Feeding

**Published:** 1916-09-23

**Authors:** 


### THE SCHOOL DOCTOR AND SCHOOL FEEDING.

The history of the agitation which led to the passing of the Provision of Meals Act, 1906, shows beyond dispute that school feeding was instituted as a means of preventing and remedying the widely prevalent physical deterioration in school children known to be due to lack of food. It was plainly meant to form part of the public medical care of the child population, and the physical need of the child is to be the primary consideration in providing meals. This point is of more than historical importance, for in many areas the purpose of the Act has been consistently misunderstood. It is regarded simply as a new and rather superfluous means of granting relief; the economic or moral condition of the parent is considered rather than the welfare of the child, physical needs are deemed irrelevant, and the school doctor is deprived of perhaps his most powerful weapon for improving the condition of his charges. Some authorities have even refused to adopt the Act at all, in spite of the fact that their attention has been repeatedly drawn by their medical officers to the presence of ill-nourished children in their schools.

Although the medical significance of school-feeding can hardly be over-emphasised, it is clear that its efficacy as a preventive of malnutrition cannot have a fair chance unless the controlling committee encourages parents to apply promptly of their own accord whenever a genuine need arises. The folly of insisting that semi-starvation must go on until the child shows signs of physical damage before it can be fed, ought to be, but is not always, self-evident. In any well-administered locality the majority of the children on the dinner-list will be put there by the teachers on application of the parents, and will never come before the school doctor at all by reason of their poor nutrition. But it is a very important part of his duties to look out for the inevitable residuum of underfed children —for example, those whose parents are too neglectful or too proud to apply for meals themselves, and to see that such children are supplied with meals or milk. The task bristles with unexpected difficulties. The signs of underfeeding in children are more elusive than is commonly supposed and may be wrongly attributed to other pathological conditions or hygienic defects. Further, many such conditions do in fact produce appearances almost identical with those due to lack of food—for example, latent tuberculosis, old rickets, lack of sleep, chronic digestive disturbance. Not infrequently the school doctor has to make the practical experiment of feeding the child provisionally and noting the result before he can offer a diagnosis of the cause of its sub-normal condition.

The dietary for the school meals should be recognised as a matter for the medical expert, and not for some enthusiastic clerk with a book-knowledge of "food values." Such nourishment as a "necessitous child" is likely to get at home will consist mainly of tea, and carbohydrates and tinned food; the school diet table should be correspondingly rich in proteids, fats, and vitamines. If the full daily allowance of proteid for a growing child is taken as 80 grams (children need relatively much more proteid than adults), the school dinner should contain at least 50 grams. Where this proteid is provided in the form of the cheap pulses (lentils, peas, and beans) the cooking needs careful supervision. They are frequently served in a hard, indigestible state, which provokes diarrhœa and vomiting, and incidentally earns for them a bad reputation among parents which prevents their proper use among the poor. Monotony is a very prevalent and quite inexcusable fault. In some areas a thick, unpalatable mess reappears daily under different aliases, until the most Spartan child is nauseated. By contrast, the admirable menus prepared by the Bradford, London, and Nottingham Committees show what can be achieved with 2½d. a day and a little forethought.

The service of the meals is an aspect of school feeding which concerns the school doctor more closely than is usually admitted. The educational possibilities of school feeding are being increasingly appreciated, but the experience of medical officers proves that too often the surroundings of the meals are still as unhygienic as they are uneducational. The use of damp and ill-ventilated cloakrooms and cellars is now almost abandoned, but bad supervision is still a serious nuisance. Little ones with filthy fingers are allowed, or even encouraged, to bolt their food; infants are seen struggling with bowls of soup at the level of their heads; and delicate children who exhibit the wretched appetites of the habitually underfed are either bullied or ignored. The duty of the school medical officer will lead him to point out that the alleviation of malnutrition implies trained and intelligent supervision of the whole business of school feeding in all its details.

